# Submucosal Inferior Turbinoplasty Using a New Continuous Suction Irrigation Method

**DOI:** 10.3389/fsurg.2017.00024

**Published:** 2017-05-08

**Authors:** Takeo Kanaya, Naoyuki Kohno

**Affiliations:** ^1^Otorhinolaryngology, Kosei Hospital, Tokyo, Japan; ^2^Kosei Hospital, Otorhinolaryngology, Kyorin University Hospital, Tokyo, Japan

**Keywords:** endoscopic, submucosal inferior turbinoplasty, inferior turbinate, inferior turbinate hypertrophy, suction irrigation

## Abstract

**Introduction:**

Numerous surgical techniques for inferior turbinate hypertrophy (ITH) have been reported, each with advantages and disadvantages. Submucosal turbinoplasty with removal of the bony component of the inferior turbinate (IT) is an excellent procedure to expand the nasal cavity. However, this procedure requires a large incision to remove the inferior turbinate bone (ITB) and is associated with hemorrhage, crust formation, and adhesion. A smaller incision would avoid such complications. We developed a continuous suction irrigation method that maintains a clear view of the limited surgical field of the IT. Only a single small incision is needed to insert the rigid endoscope. The surgeon can flush blood with continuous water flow and perform IT surgery without difficulty. We performed this method in 39 cases from January 2016 to January 2017. This video article demonstrates our new submucosal inferior turbinoplasty technique.

**Methods:**

Submucosal turbinoplasty using a continuous suction irrigation method was performed under general anesthesia. An irrigation-suction straw sheath system was used to create an underwater surgical field.

**Results:**

The ITB was removed safely with no severe complications. An expanded common nasal cavity was confirmed postoperatively on computed tomographic images.

**Conclusion:**

We resected the ITB safely using a continuous suction irrigation method without difficulty or complications. We believe that this method may become one of the best surgical options for ITH.

## Introduction

Inferior turbinate hypertrophy (ITH) is a common cause of nasal obstruction, which causes noticeable patient discomfort. The purpose of turbinate surgery is to regain adequate space inside the nasal cavity for patients to breathe through the nose without loss of nasal function. Preserving the functional inferior turbinate (IT) mucosa is important to avoid the risk of dry throat, nasal crusting, and an overly patent nasal cavity (1).

Numerous surgical techniques for ITH have been reported (2). Submucosal resection is one of the best options for ITH in reducing the bony component of the IT without severe complications. A large incision, however, is often necessary to remove the inferior turbinate bone (ITB) during submucosal inferior turbinoplasty (SIT), which is associated with hemorrhage, crust formation, and adhesion. A small incision is necessary to reduce such complications, but it reduces the surgical field of view. Hence, a new surgical procedure with a smaller incision is needed that maintains a clear surgical view in this very narrow space. Using a continuous suction irrigation method enabled us to obtain a clear surgical view and still perform fine manipulations including hemostasis and bone removal. We performed this method in 39 cases from January 2016 to January 2017 without experiencing severe complications. This video article demonstrates our new continuous suction irrigation method for SIT.

## Methods

### Irrigation-Suction Straw Sheath System

**Figure 1:**
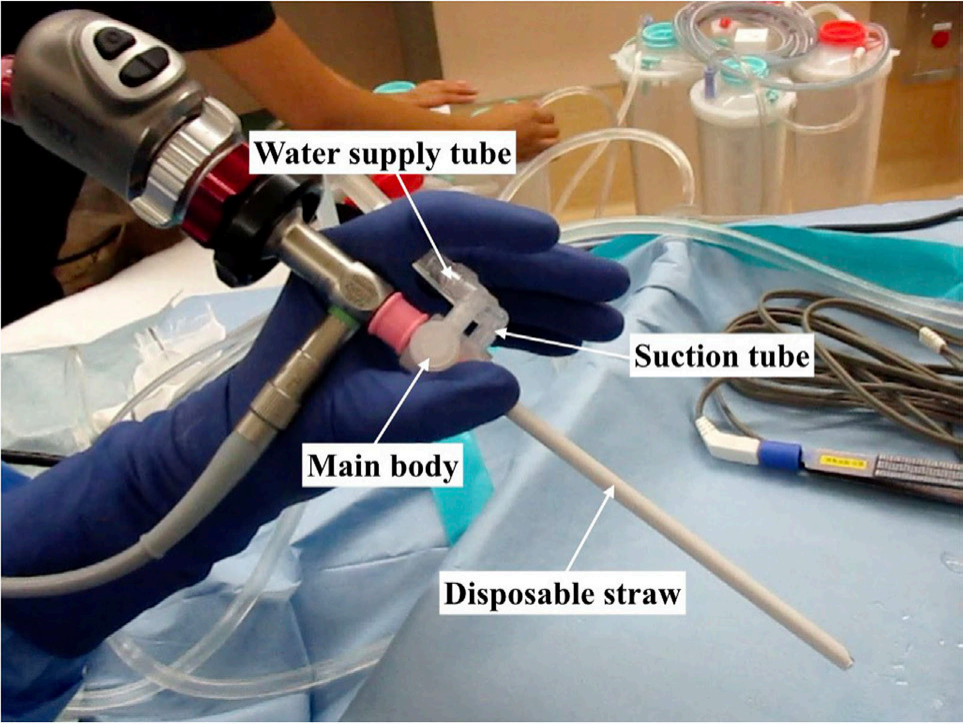
**The irrigation-suction straw sheath system consists of four parts: main body, disposable straw, water supply tube, and suction tube**.

An irrigation-suction straw sheath system (K-endosheath; Koken Co., Tokyo, Japan) is indispensable when creating a fine surgical field (3). This system has two important parts: a disposable straw and a main body. A rigid endoscope passes through both the disposable straw and the main body. A water supply tube and a suction tube are connected to the main body (Figure 1), and the main body has a push button to supply water to the disposable straw. The push button is depressed continuously to maintain a stream of water during surgery. The suction tube, a Nelaton catheter, is inserted into the opposite side of the nasal cavity to absorb water at the nasopharynx and to prevent water overflow.

### Preparation

The operation is performed under general anesthesia using a 4-mm 0° endoscope. The surgeon sits on the patient’s right side, facing the monitor. A total of 1:2,000 epinephrine-soaked gauze is used to shrink the IT mucosa, and 1:100,000 epinephrine is injected into the anterior, middle, and posterior IT.

### Surgical Technique

**Figure 2:**
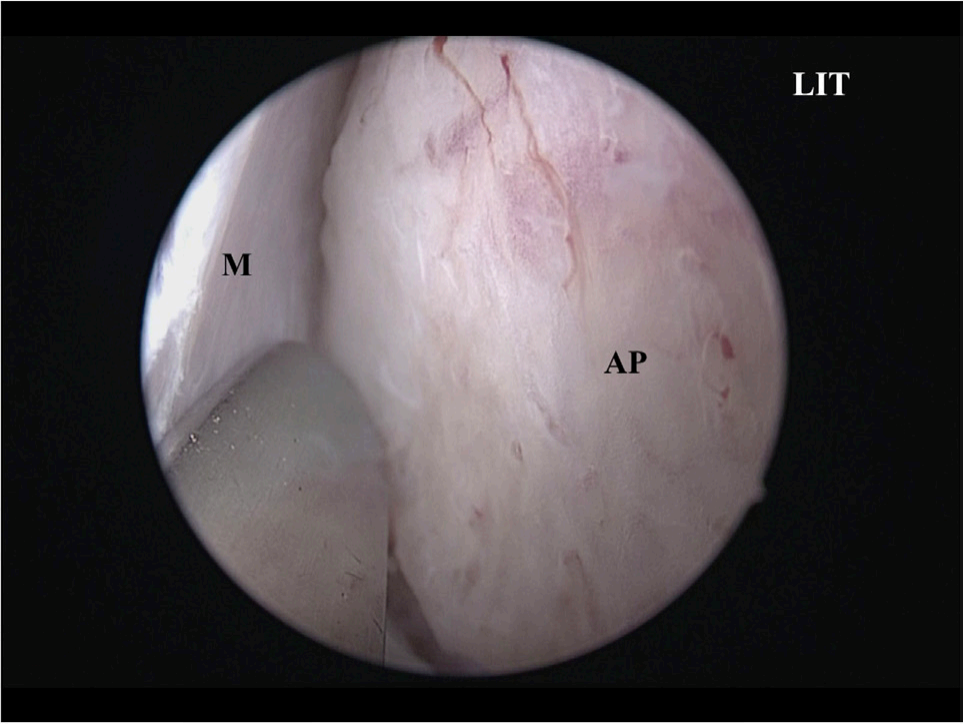
**The AP is exposed**. LIT, left inferior turbinate; AP, anterior inferior turbinate bone; M, mucoperiosteum.

**Figure 3:**
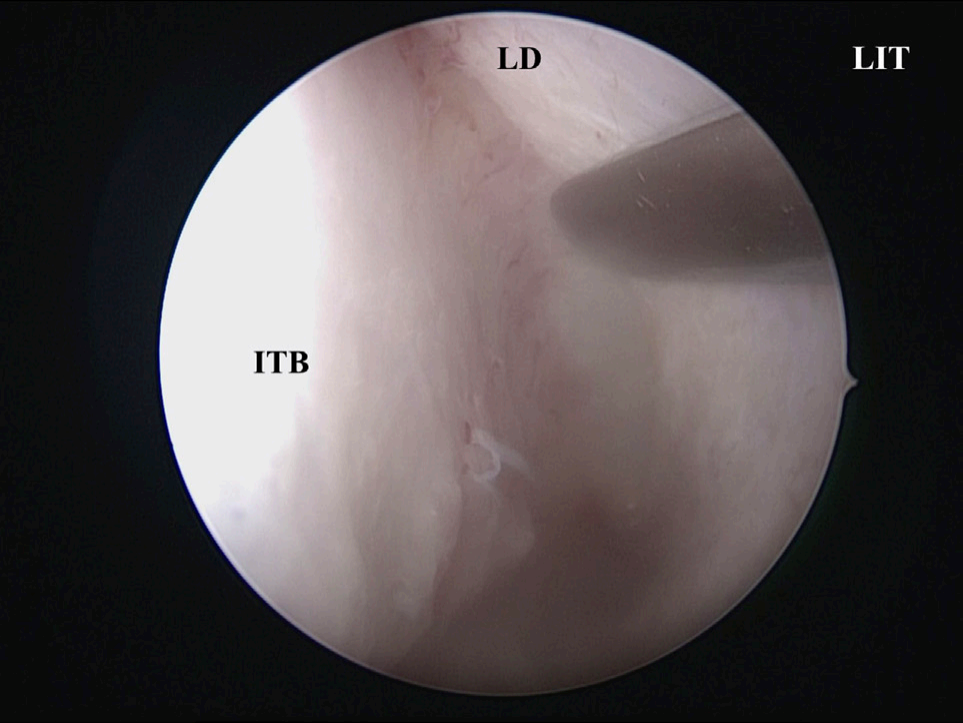
**The LD is identified at the side of the inferior meatus**. LIT, left inferior turbinate; ITB, inferior turbinate bone; LD, lacrimal duct.

**Figure 4:**
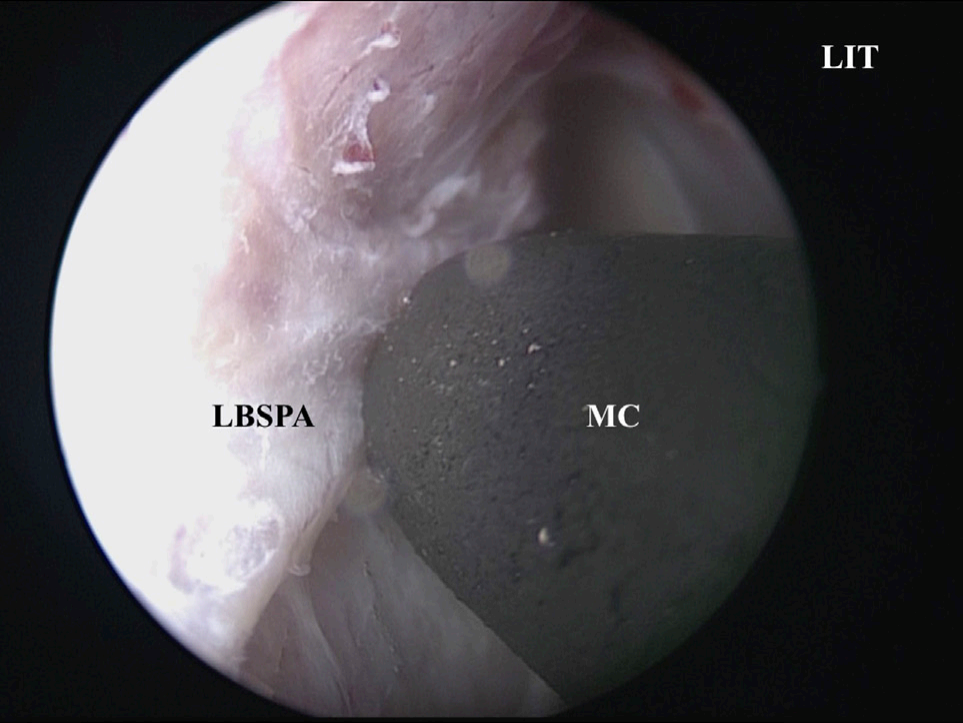
**The LBSPA is cauterized using an MC**. LIT, left inferior turbinate; MC, monopolar coagulator; LBSPA, lateral branch of the sphenopalatine artery.

A vertical incision is made at the anterior IT with a scalpel, and the anterior ITB is then exposed (Figure 2). The mucoperiosteum is elevated from the anterior to medial ITB using an elevator, and the rigid endoscope is inserted into the side of the inferior meatus. The mucoperiosteum on the inferior meatal side is separated from the ITB. At this point, the location of the lacrimal duct is confirmed (Figure 3), and the maxillary process of the IT is removed using an osteotome. Fragments of the ITB are then extracted. The lateral branch of the sphenopalatine artery is exposed at the posterior IT and then cauterized with a monopolar coagulator to reduce the possibility of hemorrhage (Figure 4). Finally, the incision is closed using 4-0 absorbable suture, and Merocel^®^ packing is placed in the common nasal meatus. The packing is removed on the first postoperative day.

## Results

**Figure 5:**
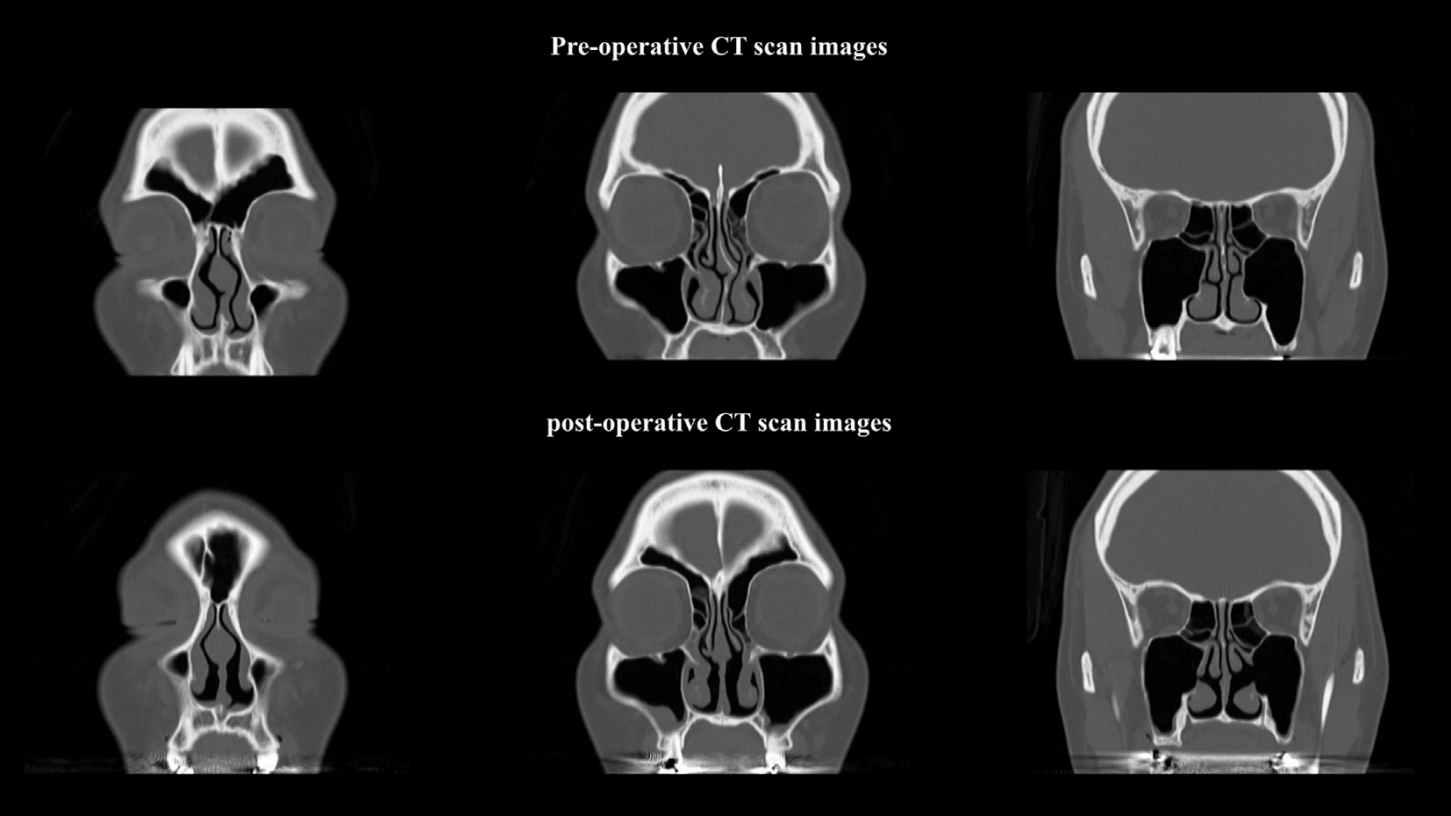
**Post-operative computed tomographic images show an expanded bilateral common nasal meatus**.

The ITB was resected without severe complications including hemorrhage, crust formation, adhesion, or loss of nasal function. To determine the effectiveness of this method, we compared areas of the common nasal meatus using computed tomography (SYNAPSE; Fujifilm, Tokyo, Japan) preoperatively and 3 months after surgery. The three areas that were measured were level areas of the anterior, middle, and posterior IT (Table 1). An expanded area of bilateral common nasal meatus accompanied by lateralization of the IT was confirmed by computed tomography (Figure 5).

**Table 1:** **Comparison of areas of the common nasal meatus**.

	Anterior		Middle		Posterior	
	R	L	R	L	R	L
Before surgery (mm^2^)	57.21	34.63	42.94	42.97	36.51	47.23
Three months after surgery (mm^2^)	108.69	134.86	96.96	157.02	69.39	93.87

## Discussion

Submucosal inferior turbinoplasty is one of the best surgical options to reduce IT volume. Recently, pyriform turbinoplasty and medial flap inferior turbinoplasty have been reported (4, 5). These techniques are useful for improving nasal patency; however, they can be associated with hemorrhage, curst formation, and adhesion because of the large surgical wound. We believe that our method improves on other techniques because it minimizes the size of the surgical wound, preserves almost all of the IT mucosa, allows hemostasis by providing access to the branch of the sphenopalatine artery, and permits fine surgical maneuvers.

This continuous suction irrigation method has several limitations. First, the surgeon must be highly skilled in endoscopic nasal surgery. To perform fine surgical maneuvers underwater, surgeons must perform fine manipulations in the narrow nasal space, which resembles a small pocket, while avoiding tears in the IT mucosa. Second, SIT may lead to atrophic rhinitis; however, this complication has not been reported in two articles evaluating the long-term results of SIT (6, 7). Similarly, we have not experienced atrophic rhinitis in our cases. We believe that preserving the IT mucoperiosteum is particularly important to avoid atrophic rhinitis after SIT, and the mucoperiosteum is readily preserved using our continuous suction irrigation method. Third, the follow-up period in our study was short. Therefore, longer follow-up periods are needed to determine the value of this method.

### Comparison with Other Surgical Technique for ITH other than SIT

A number of surgical procedures for ITH have been reported: lateralization, total and partial turbinectomy, submucosal turbinoplasty, electrocoagulation, radiofrequency, argon plasma coagulation, and laser surgery, among others (2). Which surgical procedure for ITH is the best remains to be determined. Passàli et al reported long-term results of six types of surgery for ITH: electrocautery, cryotherapy, laser cautery, submucosal resection without lateral displacement, submucosal resection with lateral displacement, and turbinectomy (7). They concluded that submucosal resection combined with lateral displacement was the first-choice operation in cases of nasal obstruction due to ITH. We, too, think that experientially SIT has better long-term results than other surgical therapies for ITH, but we still need to evaluate the long-term results. We think that the most important thing is to select the best surgical therapy for each patient.

## Conclusion

The clear surgical view provided by our continuous suction irrigation method allows SIT to be performed safely and reduces the risk of complications, including those caused by hemorrhage during SIT. We believe that our continuous suction irrigation method may be useful not only for SIT but also for other endoscopic nasal surgery.

## Ethics Statement

This surgical method was in accordance with the Ethical Guidelines for Medical and Health Research Involving Human Subjects issued by the Ministry of Health, Labor and Welfare in Japan. The institutional ethics committee in Kosei Hospital approved the method. Written informed consent was gained prior to surgery from the participants.

## Author Contributions

TK was the major contributor to writing the manuscript. NK contributed to writing and correcting the manuscript. All authors read approved the final manuscript.

## Conflict of Interest Statement

The authors declare that the study was conducted in the absence of any commercial or financial relationships that could be constructed as a potential conflict of interest.

## Abbreviations

IT, inferior turbinate; ITB, inferior turbinate bone; ITH, inferior turbinate hypertrophy; SIT, submucosal inferior turbinoplasty.
